# Molybdenum – a scoping review for Nordic Nutrition Recommendations 2023

**DOI:** 10.29219/fnr.v67.10326

**Published:** 2023-12-14

**Authors:** Agneta Oskarsson, Maria Kippler

**Affiliations:** 1 Department of Biomedical Sciences and Veterinary Public Health, Swedish University of Agricultural Sciences, Uppsala, Sweden; 2 Institute of Environmental Medicine, Karolinska Institute, Stockholm, Sweden

**Keywords:** molybdenum, molybdate, molybdenum cofactor, nutrition recommendations

## Abstract

Molybdenum is an essential element in the form of the molybdenum cofactor (Moco). In humans, Moco is required for four enzymes: xanthine oxidase (XO), aldehyde oxidase, sulfite oxidase (SO), and mitochondrial amidoxime-reducing component (mARC). The enzymes are involved in the oxidation of purines to uric acid, metabolism of aromatic aldehydes and heterocyclic compounds, and in the catabolism of sulfur amino acids. Molybdenum cofactor deficiency is a rare autosomal recessive syndrome due to a defective synthesis of Moco, resulting in a deficiency of all the molybdoenzymes. There are no reports on clinical signs of dietary molybdenum deficiency in otherwise healthy humans. Water-soluble molybdate is efficiently absorbed from the digestive tract. The body retention is regulated by urinary excretion. Plasma molybdenum reflects long-term intake and 24-h urinary excretion is related to recent intake. There are no biochemical markers of molybdenum status. Cereal products are the main contributors to molybdenum dietary intake, estimated to 100–170 μg/day in Nordic studies. Little data are available on molybdenum toxicity in humans. A tolerable upper intake level of molybdenum has been based on reproductive toxicity in rats, but the effects have not been reproduced in more recent studies. The U.S. Institute of Medicine (IOM, present National Academy of Sciences, Engineering, and Medicine; NASEM) established a Recommended Dietary Allowance of 45 μg/day in adult men and women in 2001, based on a small study reporting urinary excretion in balance with intake at 22 μg/day. The European Food Safety Authority (EFSA) considered in 2013 the evidence to be insufficient to derive an Average Requirement and a Population Reference Intake, but proposed an Adequate Intake of 65 μg/day for adults.

## Popular scientific summary

- Molybdenum is an essential trace element for humans in the form of molybdenum cofactor.
- Molybdenum cofactor deficiency is a rare genetic disorder that causes a deficiency of all the molybdenum-dependent enzymes.
- Clinical signs of dietary molybdenum deficiency have not been reported in healthy humans.
- There are no biomarkers of molybdenum status, but blood levels and 24-h urinary excretion are markers of molybdenum intake.
- The main dietary sources of molybdenum are cereal products, vegetables, and dairy products.

Molybdenum was discovered in 1778 by the Swedish chemist Carl Wilhelm Scheele. The substance was first mistaken as lead, and after realizing that it was a new element, he named it molybdenum after the Greek word “molybdos”, which means “lead-like”. Molybdenum was known to be essential for plants long before its role as a cofactor in human xanthine oxidase (XO) was discovered in 1953 (1).

Molybdenum is an essential element in the form of the molybdenum cofactor (Moco). More than 50 enzymes are known to be molybdenum-dependent. Most of them are found in bacteria. Molybdoenzymes play important roles in nitrogen fixation, the carbon cycle, and sulphur metabolism in various organisms. In humans the Moco is required for four enzymes, xanthine oxidase (XO), aldehyde oxidase, sulfite oxidase (SO), and mitochondrial amidoxime-reducing component (mARC) (2–4). The most recent discovered enzyme requiring Moco is mARC, which was identified in 2006 (5). The enzymes are involved in the oxidation of purines to uric acid, metabolism of aromatic aldehydes and heterocyclic compounds, and in the catabolism of sulphur amino acids. Even though considered as an essential element, there are no reports on clinical signs of dietary molybdenum deficiency in healthy humans.

The aim of this scoping review is to describe the totality of evidence for the role of molybdenum for health-related outcomes as a basis for setting or not setting a dietary reference value (DRV) for the Nordic Nutrition Recommendations (NNR) 2023 (Box 1).

Box 1 Background papers for Nordic Nutrition Recommendations 2023

- This paper is one of many scoping reviews commissioned as part of the Nordic Nutrition Recommendations 2023 (NNR2023) project (6).
- The papers are included in the extended NNR2023 report but, for transparency, these scoping reviews are also published in Food & Nutrition Research.
- The scoping reviews have been peer reviewed by independent experts in the research field according to the standard procedures of the journal.
- The scoping reviews have also been subjected to public consultations (see report to be published by the NNR2023 project).
- The NNR2023 committee has served as the editorial board.
- While these papers are a main fundament, the NNR2023 committee has the sole responsibility for setting dietary reference values in the NNR2023 project.

## Methods

This scoping review follows the protocol developed within the NNR2023 project (6). The literature search was performed in September 2021 using the following query in PubMed “molybdenum”[MeSH Terms] AND (“2011”[PDAT]: “3000”[PDAT]) AND review [Publication Type] AND Humans[Filter]. The search resulted in 60 hits of which 19 were selected for the review. Another search using the same query was performed in March 2022, resulting in 63 hits. In addition to results from PubMed, some specific original publications, reports from Nordic food agencies, and the three reviews/assessments (8, 30, 39) were included as sources of evidence.

## Physiology

### Chemical aspects

Molybdenum is a transition element with oxidation states from -2 to +6. The most common ones are +6, Mo(VI), and +4, Mo(IV). It does not occur naturally as a free metal, but exists in minerals in various oxidation states. Molybdenum is ubiquitous in food and water as soluble molybdates ($\text{Mo}(\text{VI}){\text{O}}_{4}{}^{2-}$).

### Biochemistry

Molybdenum is an essential trace element functioning as a cofactor for four mammalian enzymes: XO, aldehyde oxidase, SO, and mARC (2–5, 7). The enzymes require molybdenum linked with a pterin, molybdopterin, as a Moco. Moco is synthesized by a conserved biosynthesis pathway, which can be divided into four steps in eukaryotes. Defects in any gene involved in the Moco biosynthesis result in Moco deficiency, a rare recessive inborn error of metabolism and dysfunction of the four Moco enzymes (described further in the text).

## Metabolism: absorption, bioavailability, transport, storage, excretion

Water-soluble molybdate (${\text{MoO}}_{4}{}^{2-}$) is the form of molybdenum that can be absorbed from the digestive tract. Small amounts (1 mg or less) are almost completely absorbed (90–100%) (1, 8). Molybdenum incorporated in food is less bioavailable than molybdenum added to food. Bioavailability of molybdenum in intrinsically labelled cress ranged from 50 to 80% and for extrinsically labelled cress from 70 to 90% (9). Compartmental modelling studies have reported molybdenum bioavailability to be around 76–83% from a mixed diet (10, 11). Absorption from infant formula was 97.5% in 10 premature infants receiving 25 μg/kg body weight.

To exert its essential role, molybdenum needs to enter cells and be incorporated into the Moco. The mechanism of absorption and transport of molybdate in mammals is poorly understood. A molybdate transporter, similar to the molybdate transporters identified in other organisms, has been described (12). In addition, molybdate may enter cells non-specifically via the sulphate uptake system. In the blood, a small fraction of molybdate is bound to α2-macroglobulin while the main part is transported in erythrocytes.

The highest tissue concentrations of molybdenum are found in the liver and kidney. Total body molybdenum stores have been calculated to be approximately 2.2 mg in adult men consuming 121 μg/day molybdenum (11).

Absorbed molybdenum is rapidly excreted via the kidney. The body retention is regulated by urinary excretion. About 60% of the molybdenum dose was excreted via urine at a very low dietary intake of molybdenum (22 μg/day), while the proportion increased to more than 90% at high intake (467 μg/day) (13). Excretion via feces is low. Highly varying concentrations of molybdenum in breast milk are reported, ranging from 0.001 to 63 μg/L, with the highest levels reported during the first days of breastfeeding (8). No correlation between maternal molybdenum intake and breast milk levels have been found.

### Molecular functions, molecular mechanisms

**Fig. 1:**
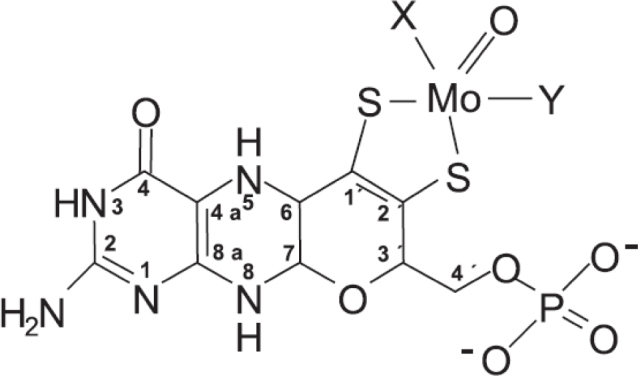
Structure of the molybdenum cofactor. Enzymes of the sulphite oxidase (SO) family. X is a single-bonded sulphur from a cysteine residue in the enzyme, and Y is a double-bonded oxygen. In enzymes of the XO family, X is a double-bonded sulphur and Y is a hydroxyl group. From Mendel, 2013, republished from Journal of Biological Chemistry under a Creative Commons license (3).

The Moco enzymes catalyze oxidation or reduction reactions, involving transfer of two electrons, which causes a change in the oxidation state of the molybdenum atom from +4 to +6 or vice versa (3, 4). There are two families of mammalian molybdenum enzymes. Sulphite oxide and mARC belong to the SO family. In the SO family, Moco is covalently bound to the enzymes via a cysteine residue (Fig. 1). SO catalyzes the terminal reaction in the degradation of the sulphur amino acids cysteine and methionine and the oxidation of sulphite to sulphate. The latest identified molybdenum-enzyme mARC, metabolizes various N-hydroxylated compounds, and has been suggested to be involved in the reduction of nitrate to NO. The native substrate of the mARC enzymes has not yet been identified. The two isoforms of mARC, which have been isolated, interact with cytochrome *b*_5_ and NADH/cytochrome *b*_5_ reductase for the enzyme activity. XO and aldehyde oxidase are structurally very similar and belong to the other family, the XO family (14). XO catalyzes the two terminal steps in purines catabolism, converting hypoxanthine to xanthine and xanthine to uric acid. Aldehyde oxidase has a broader substrate specificity than XO and is involved in the metabolism of various endogenous and exogenous N-heterocyclic compounds. Both XO enzymes produce superoxide and hydrogen peroxide, suggesting a role in cell stress response.

## Assessment of nutrition status

### Biomarkers

Plasma molybdenum reflects longer-term intake and 24-h urinary excretion is related to recent intake. In controlled human studies, plasma concentrations increased from 4 to 44 nmol/L (0.4 to 4.2 μg/L) when the intake increased from 22 to 1,400 μg/day (15). Plasma concentrations are usually <10 nmol/L (1 μg/L) (16). Geometric mean and 5^th^ and 95^th^ percentile levels of molybdenum in whole blood was 0.83 (0.44–1.78) μg/L in 1,011 individuals aged 20–91 years living in Trøndelag, Norway (17). Excretion of molybdenum in 24 h urine in 60 healthy non-smokers was studied in Sweden, and the geometric mean concentration was 54 μg/24 h (35 μg/g creatinine), with no difference between men and women (18).

Hays et al. (19) used pharmacokinetic data from controlled human studies to estimate Biomonitoring Equivalents (BEs) for molybdenum in plasma, whole blood, and urine, associated with the U.S. Institute of Medicine estimated average requirement (EAR) of 34 μg/day (20). The estimated BEs were 0.5, 0.45, and 22 μg/L in plasma, whole blood, and urine, respectively. Urinary levels expressed as μg/L were estimated to be similar to levels expressed as μg/g creatinine.

There are no biochemical markers of molybdenum status. The biochemical changes observed in subjects with genetic Moco deficiency or in the single case with molybdenum deficiency (se section Deficiency), such as low urinary and serum uric acid, elevated plasma methionine, high excretion of hypoxanthine and xanthine, and abnormal excretion of sulphur metabolites have not been observed in relation to varying intakes of molybdenum. Low activity of Moco enzymes in tissues is considered as not specific enough to determine molybdenum status, as they are also influenced by other dietary components, such as protein and amino acids.

## Dietary intake

### Dietary sources of molybdenum

Molybdenum is ubiquitous in food and water as soluble molybdates. The main dietary sources of molybdenum are cereal products, vegetables, and dairy products. The concentration of molybdenum in plants depends on soil concentration and pH. Plant uptake increases with increasing pH. Molybdenum concentration was determined in food groups of market baskets collected in Sweden in 2015 (21). Cereal products and pastries had the highest mean molybdenum concentrations of 41 and 15 μg/100 g, respectively (Table 1). Animal products and beverages had lower concentrations. Effect of cooking on molybdenum levels was investigated in a pilot study in cereal products, meat, fish, and potatoes. There were no statistically significant differences in molybdenum concentrations in raw and cooked food. Drinking water has a low concentration of molybdenum, usually below 10 μg/L.

**Table 1:** Molybdenum median concentrations in food groups and average daily intake per food group in market baskets from Sweden 2015 (21)

Food group	Mean concentration in μg/100 g; *N* = 5 *(Market Basket 2010; N = 5–14)*	Average daily per capita intake (μg) (% of total intake)
Cereal products	41 (*35*)	94 (55)
Pastries	15 (*16*)	7.3 (4.2)
Meat	5 (*3.8*)	10 (5.8)
Fish	1.0 (*1.0*)	0.52 (0.3)
Dairy products solid	7.5 (*5.7*)^a^	15 (8.7)
Dairy products liquid	4.5 (*5.7*)^a^	6.0 (3.5)
Eggs	6.0 (*5.7*)	1.7 (1.0)
Fats and oils	1.3 (*0.8*)	0.58 (0.3)
Vegetables	7.6 (*8.4*)	15 (8.7)
Fruits	3.4 (*1.8*)	8.0 (4.6)
Potatoes	5.9 (*5.8*)	7.4 (4.3)
Sugar and sweets	5.2 (4.6)	6.6 (3.8)
Beverages^b^	0.2 (0.2)	0.55 (0.3)
**Total daily intake**		172 (100)

Mean concentrations in food groups from the Market Basket in 2010 is presented in italics (26).

a Not separated into solid and liquid dairy products.

b Soft drinks, mineral water, beer (up to 3.5 vol. % alcohol).

Cow’s milk has a higher molybdenum concentration (3.4 and 4.8 μg/100 g have been reported) than mature human milk. Infant formulas, based on cow’s milk or soy, have molybdenum concentrations from 1.54 to 8.03 μg/100 mL (22). Supplementation of molybdenum to preterm infants is recommended in doses of 0.3–5 μg molybdenum/kg body weight per day if enteral, and approximately 0.25 μg molybdenum/kg body weight per day if parenteral (23).

### Dietary intake of molybdenum

There are few published studies on the dietary intake of molybdenum in the Nordic countries. Duplicate diet studies from Finland and Denmark reported mean dietary intakes of molybdenum of 112 and 100 μg/day, respectively (24). Dietary intake of molybdenum in the Danish population (4–75 years) was assessed in 2011–2013 through a 4-day food record (reported in (25). The intake estimates were calculated by the Technical University of Denmark, however considered by them to be of high uncertainty. Intake estimated medians (and 95%-iles) varied in the age groups, from 30 (45) μg/day, in the age group 1–3 years, to 61 (103) μg/day in men, 65–75 years of age. The Swedish market basket studies in 2010 (26) and 2015 (21) reported a mean daily estimated intake in adults of 157 and 172 μg molybdenum, respectively. The intake was estimated by the use of food production and trade statistics, in combination with population statistics, per capita (population mean) intakes of nutrients, and molybdenum levels in food available on the Swedish market (Table 1). Main contributors to estimated molybdenum intake were cereal products (55%), dairy products (12%), and vegetables (9%) (Table 1).

Duplicate diet studies in Germany have reported intakes of 58 μg/day, and total diet studies from Italy, France, and UK have reported intakes between 79.6 and 124 μg/day (8).

Molybdates may be added to foods and supplements, which will contribute to the intake of molybdenum.

In children, mean intakes of 74.9 μg/day (3–17 years) in France, and about 3 μg/kg body weight per day (4–18 years) and 4.8 μg/kg body weight per day (1.5–4.5 years) in the UK have been reported (8).

## Health outcomes relevant for the Nordic countries

There are no specific data on health outcomes of deficiency or toxicity in the Nordic countries.

### Deficiency

There are no reports on clinical signs of molybdenum deficiency in otherwise healthy humans. One case of a syndrome suggestive of molybdenum deficiency was reported in a patient with Crohn’s disease (27). The patient was on total parenteral nutrition lacking molybdenum for 12 months. Clinical symptoms included irritability, tachycardia, tachypnea, and night blindness. Furthermore, the patient had low plasma methionine, low serum uric acid, and reduced urinary concentrations of sulphate, thiosulphate, and uric acid. Treatment with 300 μg/day of ammonium molybdate resulted in improvement of the clinical symptoms and the biochemical parameters returned to normal after 30 days.

Molybdenum cofactor deficiency is a rare autosomal recessive syndrome due to a defective synthesis of molybdenum cofactor in the liver, resulting in a deficiency of all the molybdoenzymes in humans (4, 28). It was first described in 1978, and since then more than 200 patients have been reported with Moco deficiency (4). There are three types of the deficiency depending on which steps in the biosynthesis is disrupted. Neurological damage and seizures starting shortly after birth, are the most common symptoms, followed by feeding difficulties and usually death at an early age. The symptoms are caused by accumulated toxic levels of sulphite as a product of cysteine catabolism, due to lack of SO activity. A patient diagnosed with molybdenum cofactor deficiency at the age of 6 days, was treated with cyclic pyranopterin monophosphate intravenously. All markers of sulfite oxidase and XO deficiency returned to normal, convulsions disappeared and further neurodegeneration by toxic metabolites was stopped (29).

### Toxicity

Molybdenum toxicity has recently been reviewed (30). Little data are available on molybdenum toxicity in humans. Increased plasma uric acid and gout-like symptoms have been reported in an area with high molybdenum levels in soil, resulting in daily intakes of 10–15 mg molybdenum. Similar symptoms have been reported in subjects occupationally exposed to molybdenum. Epidemiological studies indicate an association between high plasma levels of molybdenum and accelerated decline in glomerular filtration rate.

Ruminants, such as cows and sheep, are sensitive to excessive molybdenum intake, which may lead to copper deficiency. Low molybdenum levels in feed are in contrast expected to enhance copper toxicity (31). There is a complex relationship between molybdenum, copper, and sulphur, especially in ruminants, which do not have efficient regulatory mechanisms for copper (31, 32). Sulphur and molybdenum react to form thiomolybdates, which bind strongly to copper and form insoluble copper thiomolybdates, resulting in copper depletion. This is also the background for the clinical use of tetrathiomolybdate to increase copper excretion in Wilson disease (33). Sodium molybdate dehydrate is used as a feed additive for sheep, and the safety and efficacy of the use was evaluated by European Food Safety Authority (EFSA) in 2019 (31).

In laboratory animals, excessive molybdenum may give rise to morphological and functional changes in the kidneys. Reproductive toxicity of molybdenum in rats has been reported (34) and was used as the basis to establish a tolerable upper intake level (UL) of molybdenum by the Scientific Committee for Food (SCF) in 2000 (35). The UL was set to 0.6 mg/person/day, based on reproductive toxicity in a 9-week study in rats, with 0.9 mg/kg body weight/day as no observed adverse effect level (NOAEL) and using an uncertainty factor of 100 (35). The U.S. Institute of Medicine used the same NOAEL and an uncertainty factor of 30 to derive a UL of 2 mg/person/day (20). However, reproductive toxicity has not been reproduced in more recent Organisation for Economic Co-operation and Development (OECD) guideline studies: one developmental toxicity study (36) and one two-generation reproductive toxicity study (37), where the NOAEL was 17 mg/kg body weight/day and based on systemic toxicity (30).

The Norwegian Scientific Committee for Food and Environment (VKM) assessed the dietary intake of molybdenum in relation to the UL (25). VKM evaluated food supplements with molybdenum levels of 100, 250, 500, and 1,000 μg/day against the tolerable upper limit established by SCF (35) of 0.6 mg/day. They concluded that intake of 1,000 μg/day from food supplements would not lead to exceedance of the UL for adults, while for 1–3 year old children all suggested limits would lead to exceedance. The Swedish Food Agency estimated a safe dose of molybdenum in food supplements to be 350 μg/day (38). The evaluation was based on the UL established by SCF of 0.6 mg/day (35) subtracted by an estimated high dietary intake of 250 μg/day.

## Requirement and recommended intake

Balance studies performed to establish the requirement of molybdenum in adults have been evaluated by EFSA (8). A study by Turnlund et al. (13) had very few participants but was the only one considered to be of sufficient duration to allow the body to adapt to the dietary intake and performed with constant diets under controlled conditions. Four healthy men (22–29 years old) received a diet containing 22 μg molybdenum/day for 102 days followed by 18 days on the same diet but supplemented with 467 μg molybdenum/day as ammonium molybdate. Stable molybdenum isotopes were administered during the dietary periods intravenously as ^97^Mo or orally as ^100^Mo. No changes were observed in uric acid, measured in blood and urine, or sulphite measured in urine. The balance based on dietary, urinary, and fecal molybdenum was near zero when molybdenum intake was 22 μg/day. Plasma molybdenum concentrations from this study were later used in a compartmental model of molybdenum kinetics and an intake of 43 μg molybdenum/day was estimated for maintaining the mean plasma concentration at baseline (9.4 nmol/L or 0.9 μg/L) (10).

Based on the study by Turnlund et al. (13), the U.S. Institute of Medicine in 2001 estimated the average minimum molybdenum requirement for maintaining adequate molybdenum status to be 22 μg/day. An additional 3 μg/day was added to allow for losses and an average bioavailability of 75% was used to derive an EAR of 34 μg/day, which was multiplied by 1.3 (twice a coefficient of variation of 15%) to derive a recommended dietary allowance (RDA) of 45 μg/day in adult men and women (20). An adequate intake (AI) of 3 μg/day was set for infants 7–12 months, and RDAs of 17, 22, 34, and 43 μg/day for children in the age groups 1–3, 4–8, 9–13, and 14–18, respectively. For pregnant and lactating women the RDA was set at 50 μg/day (20).

EFSA’s Panel on Dietetic Products, Nutrition and Allergies in 2013 concluded that there was insufficient evidence to derive an average requirement and a population reference intake for molybdenum (8). Due to lack of data on the relationship between molybdenum intakes and health outcomes, a DRV could not be derived. The Panel proposed an AI, based on the mean molybdenum intakes at the lower end of the range of observed intakes with mixed diets in the EU, from 10 μg/day in infants (7–11 months) to 65 μg/day in adults (≥18 years, including pregnancy and lactation) (Table 2). The AI for children were extrapolated from adult intakes using isometric scaling and the body weights of the respective age groups.

**Table 2:** Adequate intake of molybdenum for infants, children, and adults (8)

Age	Adequate Intake (μg/day)
7–11 months	10
1–3 years	15
4–6 years	20
7–10 years	30
11–14 years	45
15–17 years	65
≥18 years^a^	65

a Including pregnancy and lactation.

The NNR2012 did not include a recommendation for molybdenum intake. The evidence regarding molybdenum in relation to setting DRVs was considered limited and not sufficient to establish requirements (24). Accordingly, recommendations were not given for any age group.
